# Supplementary figures and images for: Spatio-temporal distribution of ostracod species in saline inland lakes (Mansfeld lake area; Central Germany)
Source: PeerJ. 2022 Aug 9;10:e13668. doi: 10.7717/peerj.13668 (PMC9373980; doi:10.7717/peerj.13668)

*C. torosa*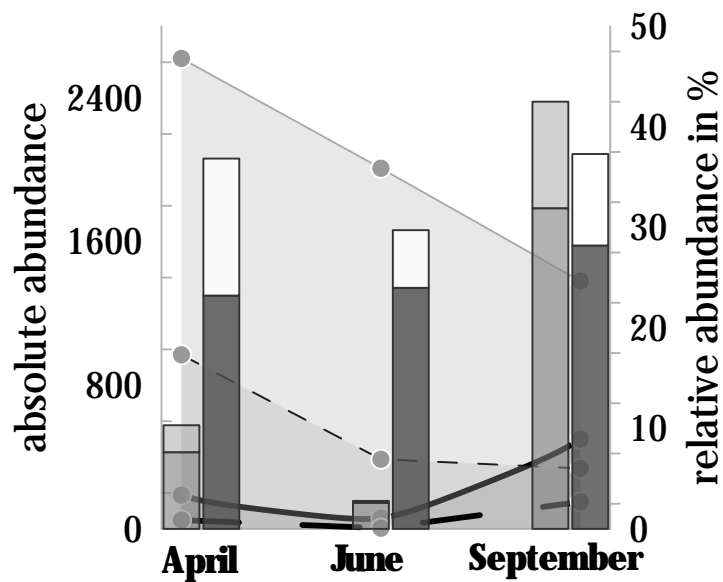*C. vidua*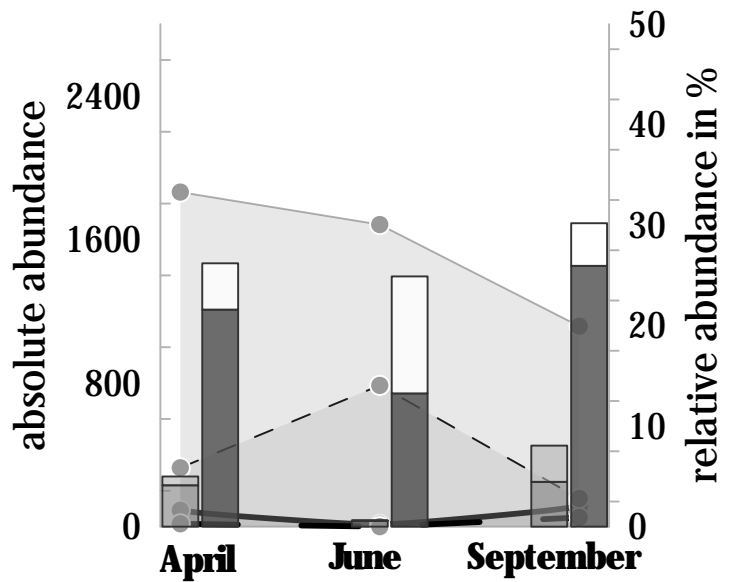*D. stevensoni*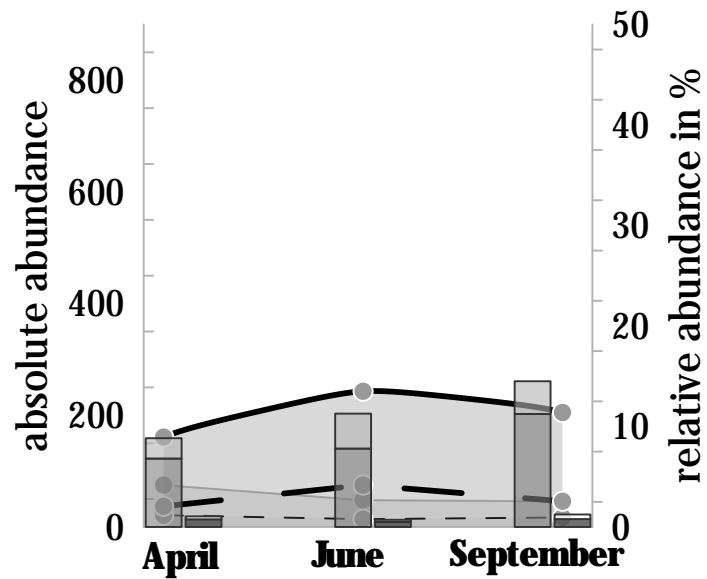*H. salina*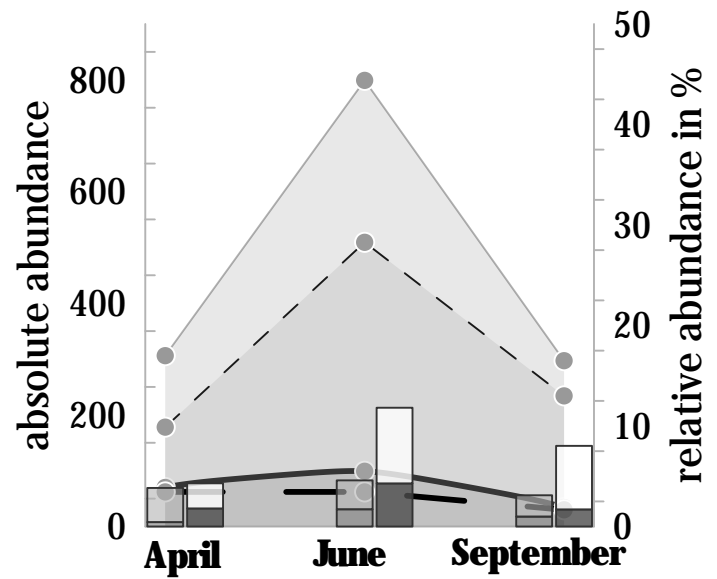*L. inopinata*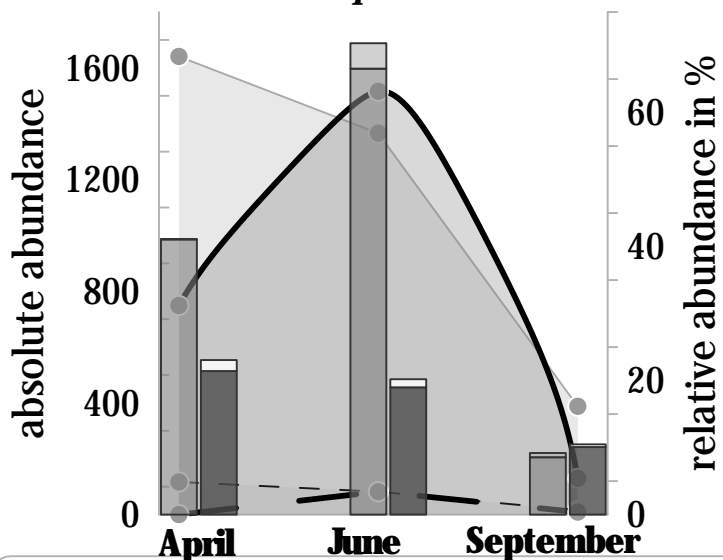*P. kraepelini*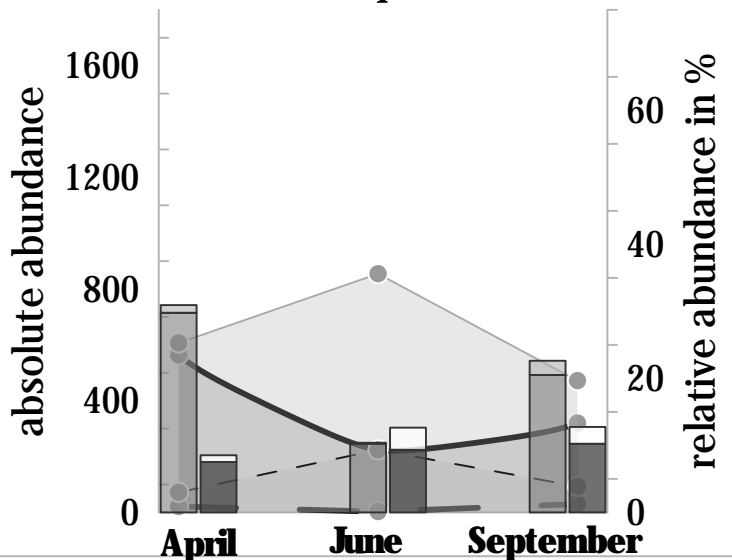

Legend

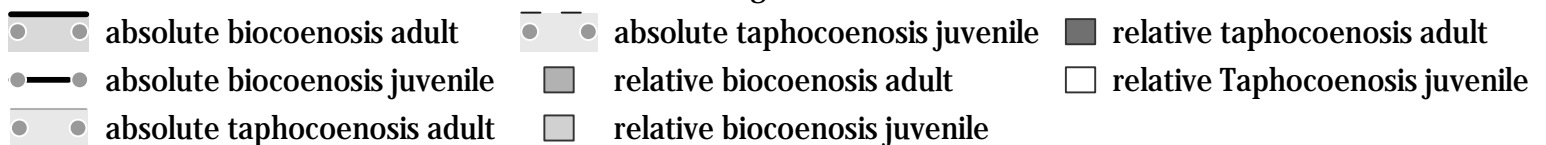

Supplement: Supplemental Information 1 [file peerj-10-13668-s001.pdf]
